# Dual function Li-reactive coating from residual lithium on Ni-rich NCM cathode material for Lithium-ion batteries

**DOI:** 10.1038/s41598-021-98123-4

**Published:** 2021-09-20

**Authors:** Tahir Sattar, Seong-Ju Sim, Bong-Soo Jin, Hyun-Soo Kim

**Affiliations:** 1 Next Generation Battery Research Center, Korea Electrotechnology Research Institute (KERI), Changwon, Republic of Korea; 2 University of Science and Technology, Daejeon, Republic of Korea; 3 Faculty of Materials and Chemical Engineering, Ghulam Ishaq Khan Institute of Engineering Sciences and Technology, Topi, Khyber Pakhtunkhwa Pakistan

**Keywords:** Batteries, Batteries

## Abstract

In this study, lithium phosphate (Li_3_PO_4_) is coated on the surface of Ni-rich LiNi_0.91_Co_0.06_Mn_0.03_O_2_ cathode material to enhance its cyclability and rate performance. The process is carried-out by achieving dual benefits, reduction of residual lithium compounds by converting them into Li_3_PO_4_ coating material. The 0.1 mol.% Li_3_PO_4_ (LiP) sample exhibits a capacity retention of 82% while the pristine NCM shows only 68.1% after 100 cycles. In addition, the LiP-0.1 NCM delivers high discharge capacities (161.9 mAh g^−1^ at 3C, 144.3 mAh g^−1^ at 4C and 94.6 mAh g^−1^ at 5C) as compared to the pristine NCM (129.3 mAh g^−1^ at 3C, 67.4 mAh g^−1^ at 4C and 33.4 mAh g^−1^ at 5C) in the voltage range of 3.0–4.3 V. In addition, the irreversible phase transition has also suppressed in the coated sample which is confirmed by cyclic voltammetry. Our study suggests that Li_3_PO_4_ coating reduces the polarization and acts as protecting layer between the electrode and electrolyte that results in the superior electrochemical performance.

## Introduction

Lithium-ion batteries (LIBs) have revolutionized the electronic industry and become the power source for electric vehicles since the first commercialization of layered oxide cathode in 1991^1–3^. Of all the LIB components, the energy density is directly determined by the cathode electrode. The commercialized cathode materials e.g. LiMn_2_O_4_ and LiFePO_4_ (< 200 Wh kg^−1^) are unable to cope up to the growing demands of high energy density^4,5^. However, Ni-rich layered oxide materials (LiNi_x_Co_y_Mn_z_O_2_; x ≥ 0.8, x + y + z = 1) have gained a lot of attention due to their high energy density and low cost^3^. The Ni-rich NCM materials e.g. LiNi_0.8_Co_0.1_Mn_0.1_O_2_ and LiNi_0.9_Co_0.05_Mn_0.05_O_2_ are most promising compositions due to their high discharge capacity (> 200 mAh g^−1^) for the energy storage system (ESS) and electric vehicles (EVs)^6,7^.

However, Ni-rich materials suffer from anisotropic volume contraction/expansion that leads to the irreversible phase transition which results in the micro-cracking of primary particles upon long cycling^8,9^. The cracking leads to the poor electrical conductivity and also destroys the solid electrolyte interphase (SEI) film which enables the penetration of liquid electrolyte into these voids of the cathode electrode^10^. Due to the consumption of Li-ions in the new surfaces of SEI results in the poor electrochemical performance of LIBs^6^. Moreover, the gas generation is another problem of LIBs practical application. It is reported that, as the bonding energy of transition metal (TM) – oxygen (O) decrease, the oxygen anions are activated due to the hybridization between TM (3d) and O (2p) orbitals. Further, the hole state in TMO_6_ octahedral which partially overlaps with the O (2p) orbital during charge–discharge process, leads to the oxygen release^11–13^. The excess lithium contents which are added before calcination process to compensate Li loss results in the formation of LiOH and Li_2_CO_3_ that contributes to increase the impedance and gas generation in the active material^1,14^. All these problems are the main culprit behind the poor electrochemical performance and hence hinder commercialization.

To address these issues, modification of active material via surface coating and doping has been effective strategies to suppress the hostile attack of electrolyte and stabilizing the host crystal lattice can enhance the electrochemical performance^15–18^. Solid electrolyte is an approach to the all-solid-state LIBs due to their non-flammability and high energy density^19–21^. Though, there are limitations due to their low conductivity and cell operation at low temperature. Surface coatings of metal oxides^22^, fluorides^23^ and phosphates^24^, Li-containing compounds^25^ has been developed as promising approach to control the particle cracking and surface reactivity of the cathode electrode^5,25,26^. Among them, Li-conductive coatings are promising candidate since they suppress the oxygen release and offers pathways to facilitate the rapid diffusion of Li-ions that leads to the enhanced electrochemical properties of NCM cathode. Feng et al.^6^ have achieved a hybrid coating of Li_3_PO_4_-AlPO_4_-Al(PO_3_)_3_ on LiNi_0.8_Co_0.1_Mn_0.1_O_2_ by reacting with Al(PO_3_)_3_. The 2-LAPO sample exhibits improved capacity retention of 85.4% after 50 cycles as compared to pristine (79.93%) at 30 °C. Raman, XRD and TEM results show that the enhanced electrochemical performance is attributed to reduced transition metal ion dissolution and lithium residue. Wang et al.^9^ have coated Li_3_PO_4_ on LiNi_0.8_Co_0.1_Mn_0.1_O_2_ by mixing LiOH, precursor and (NH_4_)_2_HPO_4_ in distilled water. The surface modification blocks the direct contact of electrolyte with active material. The 3 wt.% sample offers highest capacity retention but delivers lowest discharge capacity owing to thick electro-inactive coating of Li_3_PO_4_. Liu et al.^15^ have converted the surface lithium residue into lithium lanthanum titanium oxide (LLTO) coating on LiNi_0.6_Co_0.2_Mn_0.2_O_2_. The coating has alleviated the structural degradation that suppressed the increase in charge transfer resistance. Sim et al.^27^ has transformed the residual lithium compounds into lithium tungsten oxide (LWO) coating on LiNi_0.9_Co_0.05_Mn_0.05_O_2_. The 0.5 wt.% LWO-NCM delivers the superior electrochemical cyclability with the capacity retention of 84.6% after 80 cycles.

**Scheme 1:**
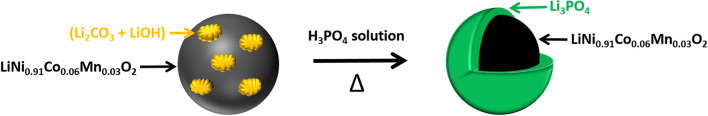
(**a**) Schematic illustration demonstrating the conversion of residual lithium into Li_3_PO_4_ coating.

In this work, an in-situ coating layer of Li_3_PO_4_ has been formed on the surface of LiNi_0.91_Co_0.06_Mn_0.03_O_2_ by one-step process without water. The reagent H_3_PO_4_ reacts with the residual lithium compounds (Li_2_CO_3_ + LiOH) and transforms them into Li_3_PO_4_ coating layer as shown schematically in the Scheme 1. This coating helped to inhibit the phase transformation, reduction of residual lithium compounds and ultimately delivers superior electrochemical performance.

## Experimental

The precursor Ni_0.91_Co_0.06_Mn_0.03_(OH)_2_ was synthesized via co-precipitation method by using aqueous solutions of NiSO_4_·6H_2_O, CoSO_4_·7H_2_O and MnSO_4_·H_2_O. NaOH solution was added as a precipitation agent and NH_4_OH solution was added as a chelating agent. The synthesized precursor powder was first mixed with LiOH.H_2_O in a molar ratio of 1:1.05. Then, the mixture was heated at 500 °C for 5 h and 680 °C for 15 h in air with the heating and cooling rates of 5 °C min^−1^ (denoted as Pristine).

For the coating process, H_3_PO_4_ (0.05, 0.1, 0.25 and 0.5 mol. %) was dissolved in ethanol and sonicated for 10 min. Later, the NCM powder was added into the above solution and stirred at 300 rpm for 5 h. During stirring, the solution was heated at 80 °C till all the ethanol evaporated. Later, the product was dried in vacuum oven at 80 °C for 10 h. The powder was heated at 500 °C for 5 h in air and finally Li_3_PO_4_ coated NCM powder was obtained. The coated samples are denoted as LiP-0.05, LiP-0.1, LiP-0.25 and LiP-0.5. The chemical reaction involved can be expressed as reaction (1–2),

1 $$ {\text{H}}_{{3}} {\text{PO}}_{{4}} + {\text{ 3LiOH }} \to {\text{ Li}}_{{3}} {\text{PO}}_{{4}} + {\text{ 3H}}_{{2}} {\text{O}} $$

2 $$ {\text{2H}}_{{3}} {\text{PO}}_{{4}} + {\text{ 3Li}}_{{2}} {\text{CO}}_{{3}} \to {\text{ 2Li}}_{{3}} {\text{PO}}_{{4}} + {\text{ 3H}}_{{2}} {\text{O }} + {\text{ 3CO}}_{{2}} $$

To support the current experimental work, Li_3_PO_4_ powder was prepared to confirm the presence of Li_3_PO_4_ coating on NCM surface. First, phosphoric acid was sonicated in ethanol for 10 min and later lithium hydroxide was added. The solution was magnetically stirred at 80 °C till all the ethanol was evaporated.

The cathode electrode was prepared by mixing the active material (A.M) and super P (CM; conducting material) in N-Methyl pyrrolidinone (NMP; solvent) at 1800 rpm for 09 min in THINKY mixer. Then, polyvinylidene fluoride (B.M; PVDF binder) was added and mixed at 1800 rpm for 06 min in THINKY mixer. The A.M, C.M and B.M was mixed in 96:02:02 wt.% ratio. The mixture was coated on Al sheet (16 μm thickness) and dried in vacuum oven at 80 °C for 10 h. Later, the coated sheets were hot-rolled at 95 °C. The loading level of cathode electrode was maintained at 15 ± 0.5 mg cm^−2^ to achieve the industrial requirement. Later, the cathode electrode was cut into 14 mm diameter and dried in vacuum oven at 120 °C for 10 h. Li-metal disc (500 μm thickness, 16 μm diameter), polyethylene film as a separator (20 μm thickness) and 1 M LiPF_6_ solution in ethylene carbonate, dimethyl carbonate, and ethyl methyl carbonate (EC:DMC:EMC = 1:1:1, v/v/v) as an electrolyte were used to assemble the coin cells in Ar-filled glove box.

The crystal structure of the samples was characterized by Powder X-ray diffraction (XRD, Philips, X-pert PRO MPD) with Cu Kα in the 2θ range of 10° to 90°. The surface valance states of elements were examined by X-ray photoelectron spectroscopy (XPS, Thermo scientific, K-alpha +). The surface morphology was analysed by Field emission scanning electron microscopy (FESEM, Hitachi S-4800), Field emission transmission electron microscopy (FETEM, Hitachi HD-2300A) and the energy dispersive X-ray detector (EDX, X-maxN, HORIBA) was used for the elemental distribution. The electrochemical testing was carried out using electrochemical equipment (Won A-Tech, WBCS 3000L) in the voltage range of 3.0–4.3 V at 25 °C. The cyclic voltammetry test was measured on a tri-electrode pouch cell by Bio-Logic workstation (VMP3) in the voltage range of 3.0–4.3 V. The counter and reference electrodes were consisting on lithium foil attached on copper foil and the working electrode. The impedance spectra were obtained by Bio-logic equipment (VSP-300) after 100 cycles in the frequency range of 1 MHz–10 mHz with 10 mV signal amplitude.

## Results and discussion

**Figure 1:**
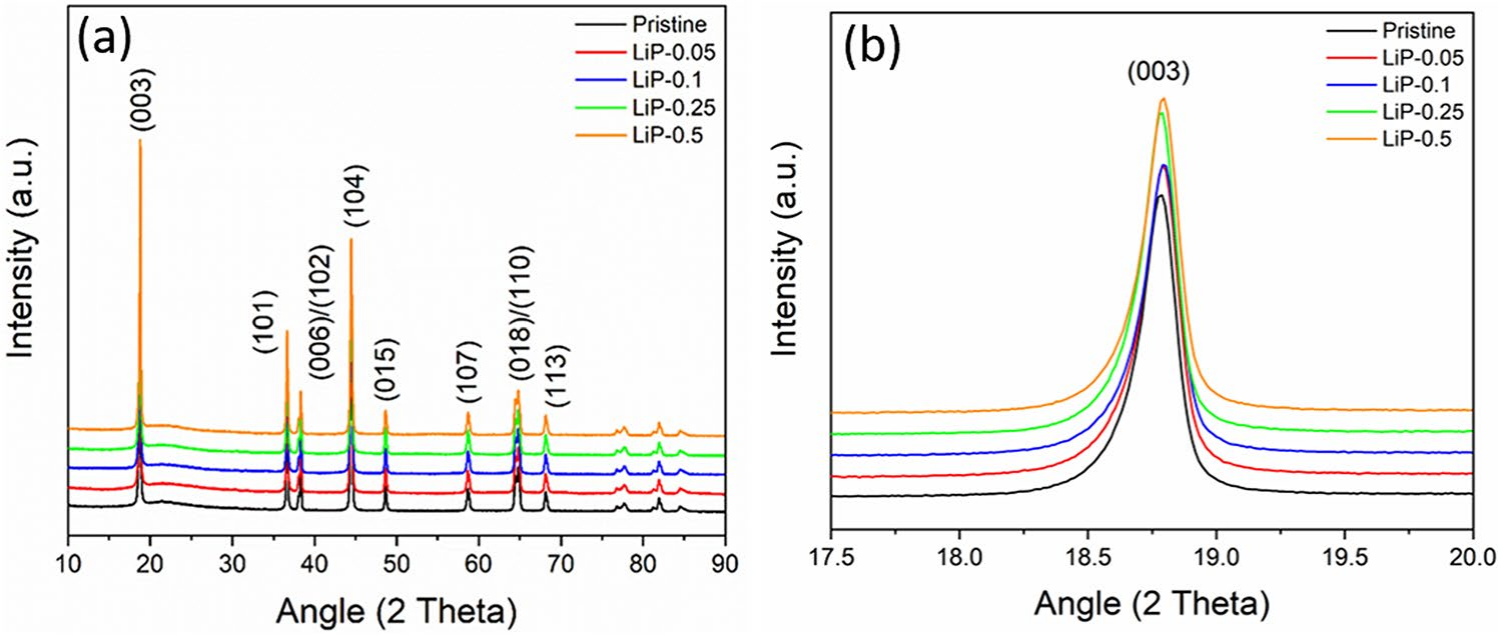
(**a**) XRD diffraction patterns of pristine and coated samples and (**b**) Magnified view of (003) reflection.

The XRD patterns of pristine and coated samples are shown in Fig. 1a. The main diffraction peaks (003) and (104) of the patterns are in good agreement with the α-NaFeO_2_ (R-3 m space group)^28^. There is no peak shift found in (003) reflection of the coated samples as shown in Fig. 1b which infers that Li_3_PO_4_ is coated on the NCM surface. The XRD pattern of synthesized Li_3_PO_4_ powder is compared with JCPD card # 98-005-0058 as shown in figure S1. All the major peaks are indexed with lithium phosphate which validates the presence of Li_3_PO_4_ coating on NCM surface. Moreover, the clear splitting of (006)/(102) and (018)/(110) pairs endorses the well-developed hexagonal layered structure in the synthesized samples as shown in figure S2a–b^9^. The intensity ratio (I_003_/I_104_) provides the information not only about the electrochemical reactivity of cathode electrode but also degree of cation mixing^28^. The similarity in the ionic radii of Li^+^ (0.76 Å) and Ni^2+^ (0.69 Å) leads to the displacement of these ions from 3a to 3b sites and a value of less than 1.2 indicates the severe cation mixing^9^. The coating of Li_3_PO_4_ can increase the cation mixing by consuming the Li atoms from the Li-slab during the reaction with coating material. However, it is worth noting that all the samples maintained low cation mixing as shown in Table 1. This confirms that only residual lithium compounds (Li_2_CO_3_ + LiOH) have participated in the reaction for forming Li_3_PO_4_ coating. The Li_3_PO_4_ peak was not indexed in the XRD pattern of coated samples due to the very small amount of the coating material.

**Table 1:** I_003_/I_104_ ratio of pristine and LiP-NCM cathode samples.

Sample	Pristine	LiP-0.05	LiP-0.1	LiP-0.25	LiP-0.5
I_003_/I_104_	1.44	1.45	1.46	1.45	1.44

**Figure 2:**
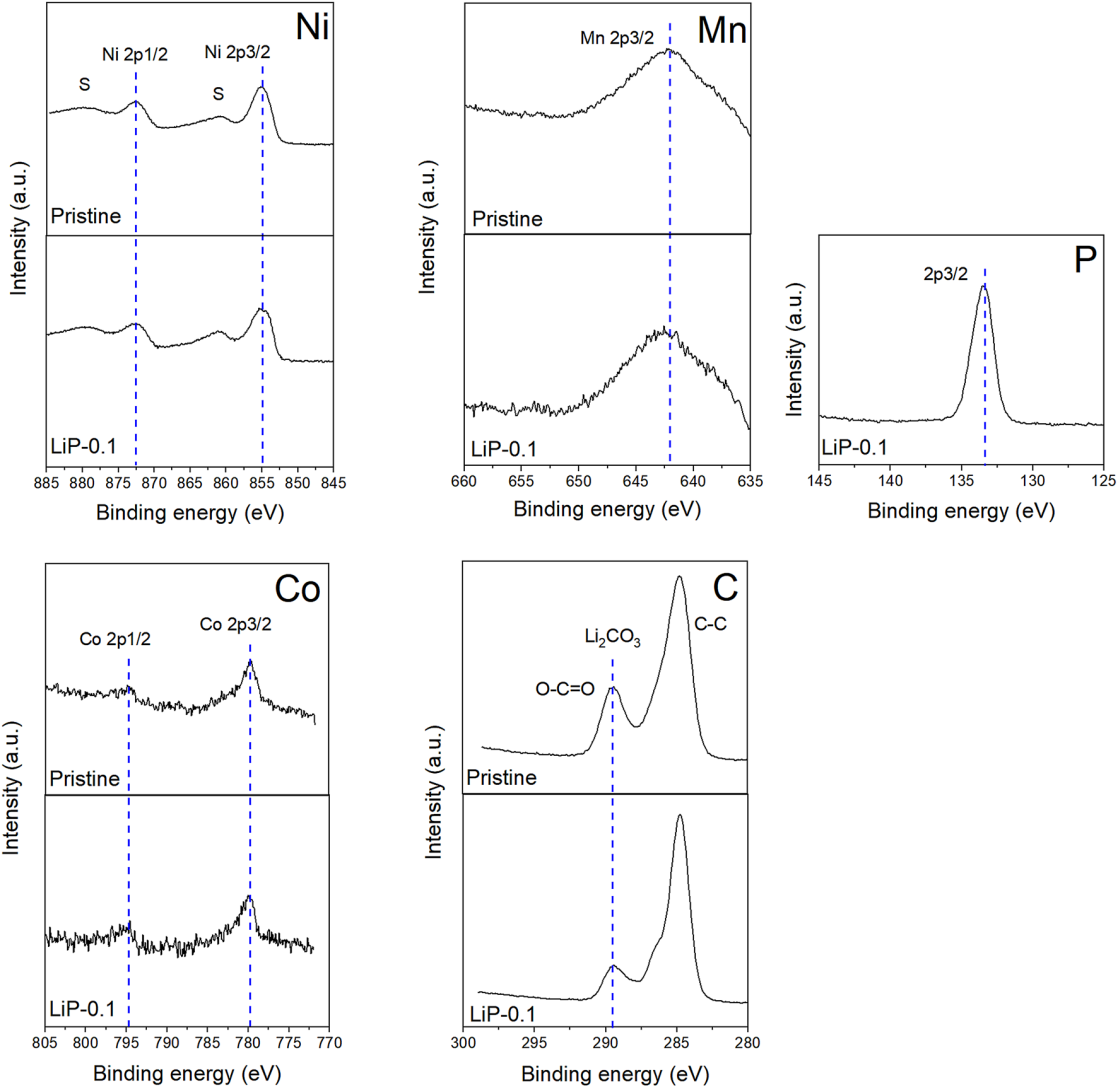
XPS spectra of Ni, Co, Mn, C and P for the pristine and LiP-0.1 sample.

X-ray photoelectron spectroscopy (XPS) was employed to investigate the oxidation states of elements for the synthesized samples as shown in figure S3. Figure 2 presents the spectra of coated samples that confirm the presence of Li, Ni, Co, Mn, C and P. The Ni 2p_3/2_ and Ni 2p_1/2_ peaks are observed at 855.11 eV and 872.6 eV in the Ni 2p spectra, respectively. The peaks of Co 2p of Co^3+^ are observed at 779.5 eV and 794.7 eV which belongs to the Co 2p_3/2_ and Co 2p_1/2_, respectively^8^. The Mn 2p_3/2_ peak is observed at 642.1 eV corresponding to the Mn^4+^^27^. The C 1 s spectrum shows suppression in the peak at 289.4 eV which is due to the lower concentration of Li_2_CO_3_ on the surface of LiP-0.1 sample as compared to pristine. This infers that the residual lithium is utilized in the formation of Li_3_PO_4_ coating material^6^. Furthermore, Fig. 2 shows the spectra of P 2p_3/2_ at 133.4 eV which is only detected in the LiP-0.1 sample due to the presence of Li_3_PO_4_ coating^6^. This confirms that Li_3_PO_4_ coating is formed on the surface of LiP-0.1 sample due to the reaction between residual lithium and phosphoric acid.

**Figure 3:**
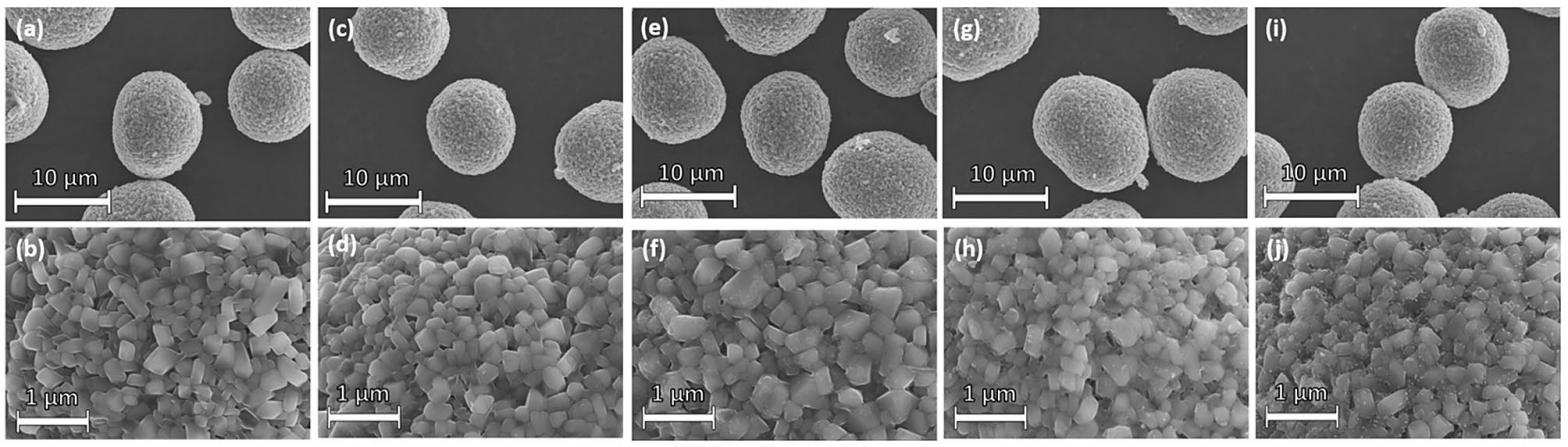
FESEM images of (**a**,**b**) Pristine, (**c**,**d**) LiP-0.05, (**e**,**f**) LiP-0.1, (**g**,**h**) LiP-0.25 and (**i**,**j**) LiP-0.5.

**Figure 4:**
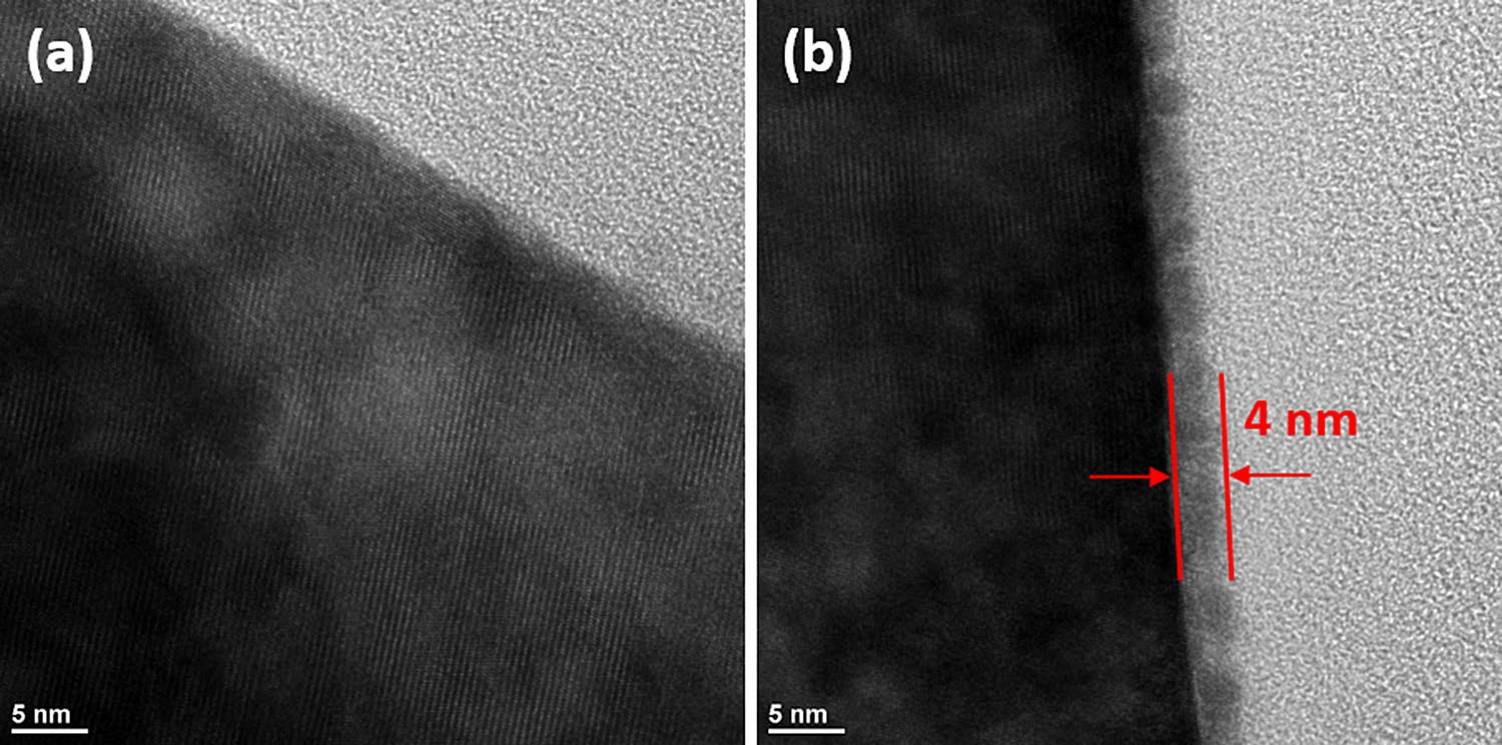
FETEM images of (**a**) Pristine and (**b**) LiP-0.1.

The surface morphology of the synthesized samples is shown in Fig. 3. All the samples show characteristic spherical morphology with average particle diameter of about 10–12 μm which are composed of nano sized primary particles (200–300 nm). The surface of pristine sample is smooth and clean. However, in case of Li_3_PO_4_ coated samples the surface becomes shiny and wetted. When the coating material reached to 0.5 mol. %, the surface is covered with small particles. The high magnification FESEM images (figure S4) reveal that the primary particles are well connected and a very thin layer of Li_3_PO_4_ coating has covered the surface of LiP-NCM samples. To further characterize the Li_3_PO_4_ coating FETEM was employed. Figure 4a,b shows the images of pristine and LiP-0.1 sample. The pristine NCM sample demonstrates a crystal structure with the lattice fringes extended to the edges. However, LiP-0.1 NCM sample shows a uniform and smooth coating layer of 4 nm as can be seen in Fig. 4b. This thin and uniform coating of Li_3_PO_4_ can protect the cathode material from the side reactions with the electrolyte and HF attack which enhance the cyclability of electrodes^9^. The presence of coating material is indexed by EDX mapping as shown in figure S5. It can be seen that phosphorus is uniformly distributed on the particles surface. FESEM, FETEM and XPS characterization techniques indicate that the NCM cathode is homogeneously coated with Li_3_PO_4_ material.

**Figure 5:**
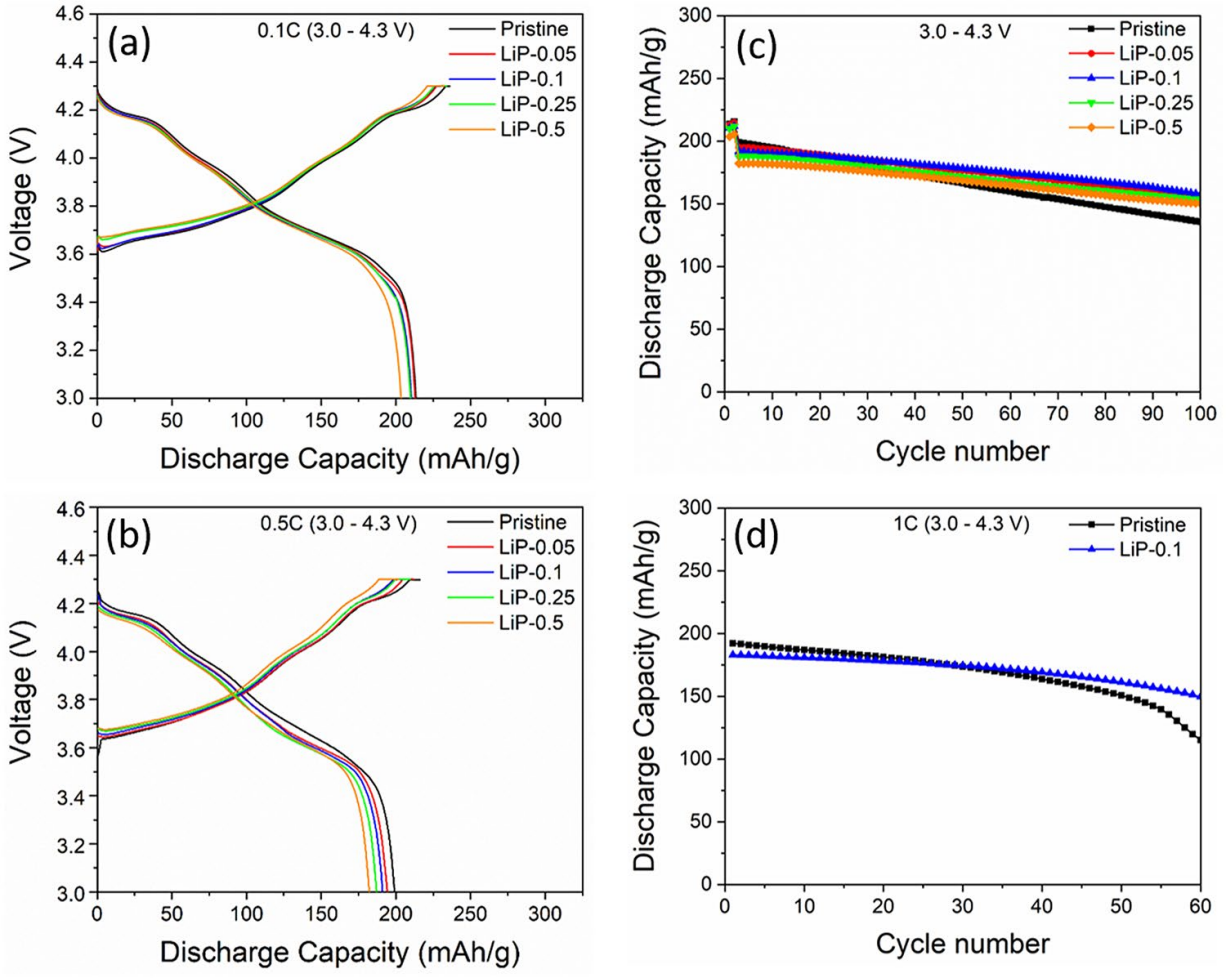
The initial charge–discharge curves of samples at (**a**) 0.1C, (**b**) 0.5C, (**c**) Cycle performance at 4.3 V and (**d**) Cycling performance of pristine and LiP-0.1 at 1C rate.

The initial charge–discharge profiles of the pristine and coated samples are presented in Fig. 5a,b. The samples were cycled at 0.1 C (02 cycle) and 0.5 C (98 cycle) in the voltage range of 3.0–4.3 V at room temperature. The charge–discharge and coulombic efficiency of 1st cycle is summarized in Table 2. All the samples exhibit similar charge–discharge curves which indicates that Li_3_PO_4_ coating does not alter the Li-ion de/lithiation in the cathode electrode. The pristine delivered the highest discharge capacity (213.5 mAh g^−1^) at 0.1C. The LiP-0.05, LiP-0.1 and LiP-0.25 showed almost similar discharge capacity, however, LiP-0.5 showed a decrease in initial discharge capacity due to the increase in thickness of non-electrochemical active coating^6,9^. In addition, the columbic efficiency of the pristine is lowest due to the electrolyte decomposition and SEI formation during first cycle as shown in Table 2. However, the Li_3_PO_4_ coating has protected and inhibits the NCM active material from the side reactions with the liquid electrolyte.

**Table 2:** Electrochemical capacities and retention of pristine and LiP-NCM cathode samples.

Sample	0.1C (1st cycle) Charge capacity (mAh g^−1^)	0.1C (1st cycle) Discharge capacity (mAh.g^−1^)	1st cycle Columbic efficiency (%)	Capacity retention after 100th cycles (%)
Pristine	239.1	213.5	89.2	68.1
LiP-0.05	233	212.7	91.2	79.5
LiP-0.1	229.3	210.4	91.7	82
LiP-0.25	228.6	209.8	91.7	82.1
LiP-0.5	222	203.5	91.6	82.4

The cycling performance of the pristine and LiP-NCM are shown in Fig. 5c. The LiP-NCM samples showed significant improvements in the cyclability as compared to the pristine, especially 0.1 mol. % LiP-NCM sample. The capacity of the pristine rapidly decreased from 199.1 mAh g^−1^ to 135 mAh g^−1^ after 100 cycles with the capacity retention of 68.1%. The rapid capacity fading is a common problem with the Ni-rich NCM cathode materials due to the above mentioned issues as reported in literature^3,5,29^. Meanwhile, the capacity retention of LiP-NCM samples are, 79.5% (LiP-0.05), 82% (LiP-0.1), 82.1% (LiP-0.25) and 82.4% (LiP-0.5). The increase in capacity retention by Li_3_PO_4_ coating is due to three major factors; (i) During the coating process, the residual lithium compounds (Li_2_CO_3_ + LiOH) are consumed to form Li_3_PO_4_ layer, (ii) The coating of NCM cathode material isolates it from excessive SEI growth by inhibiting the direct contact between cathode and electrolyte that suppress the oxygen evolution and HF attack, (iii) In addition, the bond dissociation energy of P=O (ΔH_f298_ = 596.6 kJ mol^−1^) is greater than the Ni–O (ΔH_f298_ = 391.6 kJ mol^−1^), Co–O (ΔH_f298_ = 368 kJ mol^−1^) and Mn–O (ΔH_f298_ = 402 kJ mol^−1^)^30^. All these factors enable the superior electrochemical performance of LiP-NCM cathode electrodes. Figure 5d shows the cycling stability of pristine and LiP-0.1 electrodes between 3.0–4.3 V at 1C. The pristine electrode shows higher discharge capacity but it drops from 192.3 mAh g^−1^ to 115.2 mAh g^−1^ after 60 cycles. However, LiP-0.1 capacity falls from 183 mAh g^−1^ to 149.8 mAh g^−1^ after 60 cycles. Therefore, the LiP-0.1 delivers superior cyclability of 81.8% while pristine shows only 60%.

**Figure 6:**
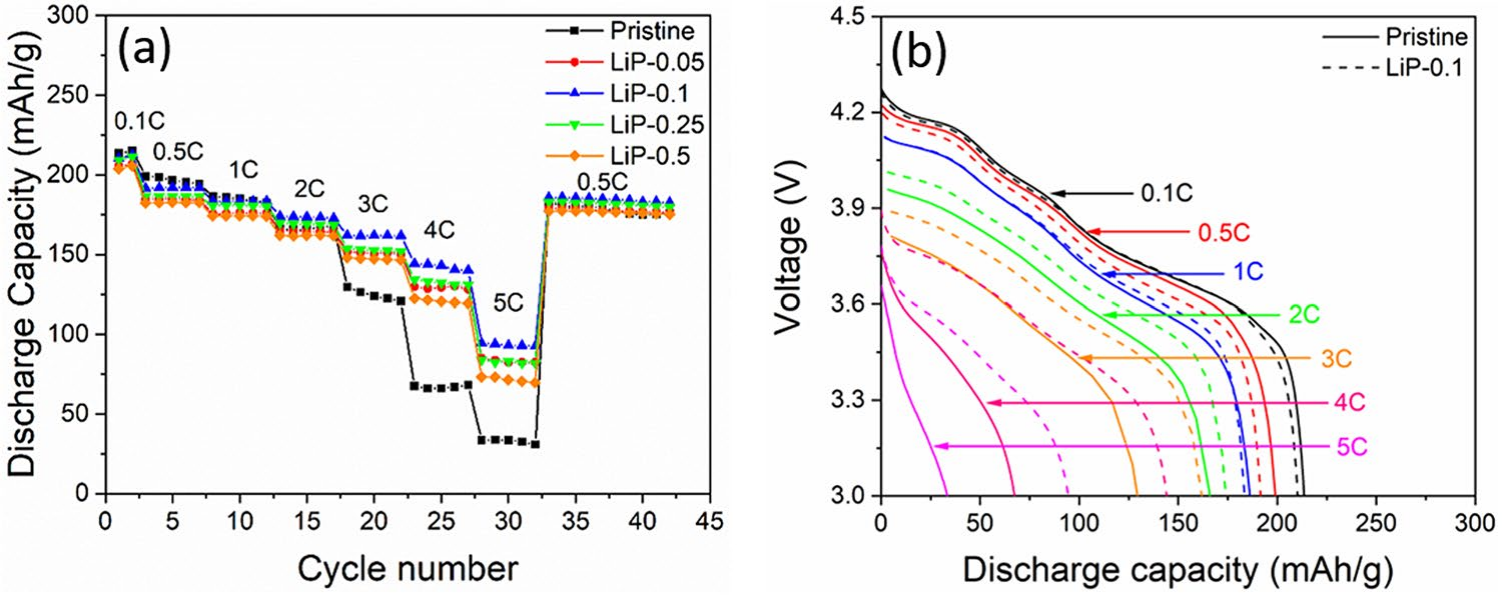
(**a**) Rate capability of synthesized samples and (**b**) Corresponding discharge curves of pristine and LiP-0.1 at various C-rates.

The rate performance of the samples was measured from 0.1C to 5C between 3.0–4.3 V as shown in Fig. 6a. All the samples show gradual decrease in the discharge capacity with the increasing C-rate due to the polarization. The pristine sample exhibited worst rate performance as the discharge rate is increased. This happens because of the direct contact between the active material and electrolyte which would demolish the particle surface structure and increases the charge transfer resistance. On the other hand, LiP-NCM electrodes exhibit superior discharge capacity, particularly LiP-0.1 delivers highest discharge capacity (94.5 mAh g^−1^) than the pristine (33.4 mAh g^−1^) at 5C. It is reported that both the electronic and ionic conductivity plays a critical role in the rate capability^31^. The residual lithium compounds on the surface of NCM material are poor conductor which impedes the Li-ions migration during cycling that leads to the increase in polarization^32^. The voltage drop in the pristine sample is increases with the C-rate than LiP-0.1 as shown in Fig. 6b. For pristine sample the voltage drop at 3C, 4C and 5C are 0.49 V, 0.53 V and 0.65 V while for LiP-0.1 is 0.42 V, 0.41 V and 0.52 V, respectively. Therefore, by converting those residual lithium compounds into ionic conductive Li_3_PO_4_ material is beneficial for the Li-ion transport^25,33^.These results confirm that LiP-NCM cathode materials maintains structural stability even at high current rate cycling.

**Figure 7:**
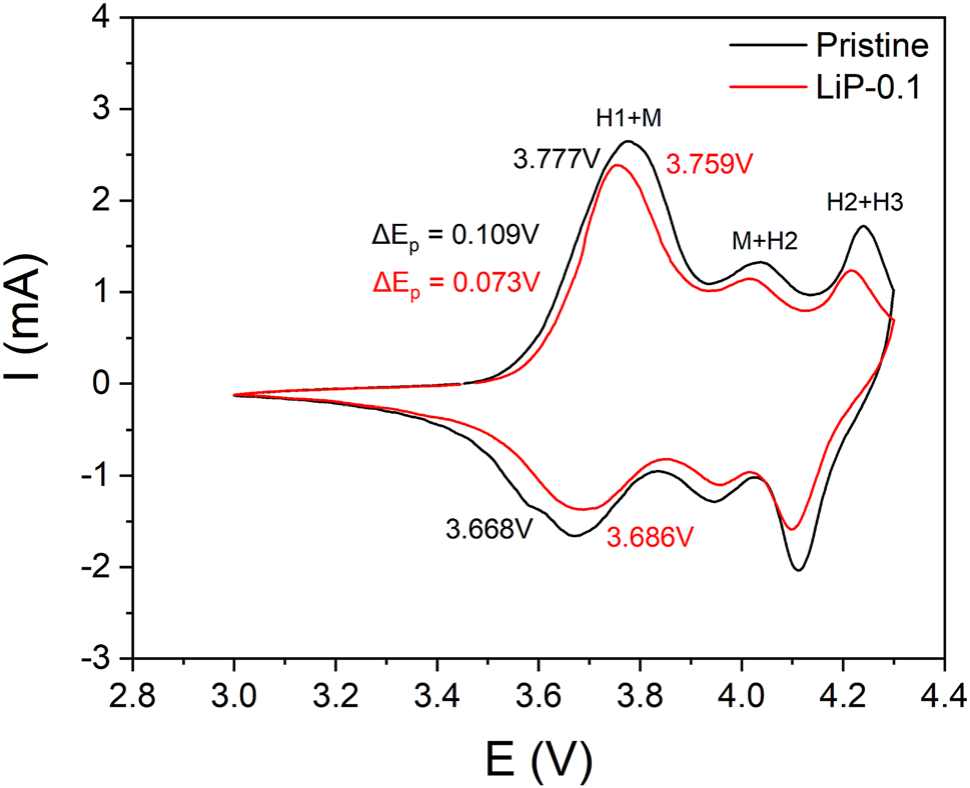
Cyclic Voltammograms of pristine and LiP-0.1.

To have better understanding of the structural stability in pristine and LiP-0.1 sample, cyclic voltammetry was carried out as shown in Fig. 7. Both the samples show a couple of redox peaks during Li^+^ de/intercalation in the range of 3.0–4.3 V, which are labelled as H1 to M (hexagonal to monoclinic), M to H2 (monoclinic to hexagonal) and H2 to H3 (hexagonal to hexagonal)^34^. The difference between the oxidation and reduction potential (△E = E_oxidation_—△E_reduction_) indicates the degree of electrode polarization^35^. Therefore, LiP-0.1 sample shows significantly less △E compared to the pristine which validates that the Li_3_PO_4_ coating reduced the polarization of NCM material. In addition, the intensities of H1 to M, M to H2 and H2 to H3 phase transitions are also suppressed in LiP-0.1 sample (as shown in Fig. 7) which leads to inhibit the particle pulverization in the NCM cathode, hence superior electrochemical performance^36^.

**Figure 8:**
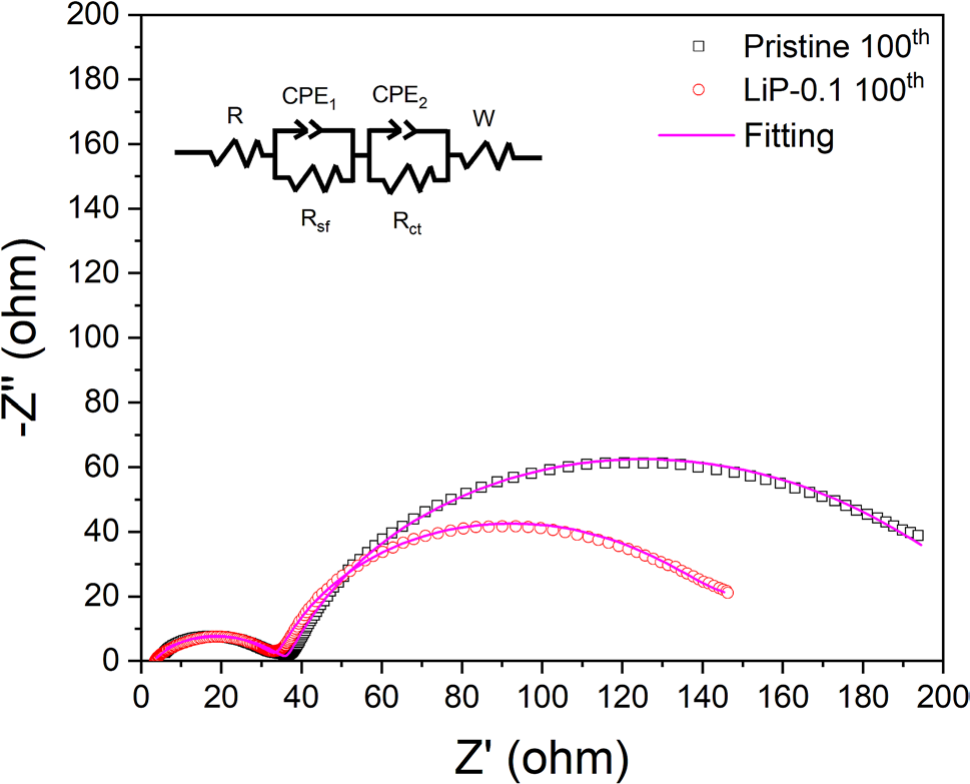
Impedance spectra with curve fittings of pristine and LiP-0.1.

Electrochemical impedance spectroscopy was carried out to further investigate the reason behind the difference in electrochemical performance of pristine and LiP-0.1 sample. The Nyquist curves are obtained after 100 cycles and fitted to an equivalent circuit as shown in the inset of Fig. 8. In the equivalent circuit, the solution resistance (R_s_) is assigned to the electrolyte solution resistance of working electrode, the first semicircle in high frequency region is related to the surface film resistance of Li-ion diffusion due to the solid electrolyte interphase (R_sf_), semi-circle in the mid frequency region is charge transfer resistance (R_ct_) which is attributed to interface between electrode and electrolyte, and sloped line at low frequency corresponds to the Warburg impedance (Z_w_) is ascribed to the Li^+^ diffusion process in bulk^9^. The LiP-NCM shows lower R_sf_ and R_ct_ values compared to pristine. The R_sf_ and R_ct_ values for pristine NCM are 35 Ω and 166.7 Ω, however, in case of LiP-0.1 are 30.8 Ω and 106.6 Ω. These results demonstrate that Li_3_PO_4_ coating inhibits the side reactions between active material and electrolyte which restricts the dissolution of transition metal ions and protects the NCM material from HF attack and also conduces the electron transfer^25^. In addition, the enhanced electrochemical performance of LiP-NCM is attributed to the Li_3_PO_4_ coating which offers superior Li^+^ conduction. The EIS results are consistent with the rate performance as shown in Fig. 6.

## Conclusions

In this work, a solvothermal coating process has been successfully carried out by converting the residual lithium compounds on the NCM surface into Li_3_PO_4_ coating material without any contact with water. Thin coating of Li^+^ conductive Li_3_PO_4_ on NCM protects it from the side reactions with the electrolyte and HF attack, while improving the Li^+^ migration, that has been confirmed by electrochemical cycling, CV and EIS analysis. The LiP-0.1 NCM provides best capacity retention of 82% after 100 cycles and superior rate capability at high C-rates. The CV results confirms that the coating has suppressed the phase transformation during Li^+^ de/intercalation. EIS results show that Li_3_PO_4_ coating has reduced the polarization and enhanced the ionic conductivity. We can conclude that; the current facile and simple coating process can ameliorate the current problems of Ni-rich cathode material to enhance their electrochemical performance.

## Supplementary Information

Supplementary Information.
