# Supplementary material for: Dual function Li-reactive coating from residual lithium on Ni-rich NCM cathode material for Lithium-ion batteries
Source: Sci Rep. 2021 Sep 20;11:18590. doi: 10.1038/s41598-021-98123-4 (PMC8452671; doi:10.1038/s41598-021-98123-4)

Supplementary information

**Dual function Li-reactive coating from residual lithium on Ni-rich NCM cathode material for Lithium-ion batteries**

Tahir Sattar ^a, b, c^, Seong-Ju Sim ^a^, Bong-Soo Jin ^a^, Hyun-Soo Kim ^a, *^

^a^Next Generation Battery Research Center, Korea Electrotechnology Research Institute (KERI), Changwon, Republic of Korea.

^b^University of Science and Technology, Daejeon, Republic of Korea.

^c^Faculty of Materials and Chemical Engineering, Ghulam Ishaq Khan Institute of Engineering Sciences and Technology, Topi, Khyber Pakhtunkhwa, Pakistan.

**^*^Corresponding author’s email:** [hskim@keri.re.kr](mailto:hskim@keri.re.kr%20)

**Tel: +82-55-280-1699 Fax: +82-55-280-1590**

**List of Figures**

Figure S1 Comparing the XRD diffraction pattern of synthesized Li_3_PO_4_ and JCPD card # 98-005-0058

Figure S2 XRD diffraction patterns of (a) (006)/(102) and (b) (018)/(110) diffraction peaks

Figure S3 X-ray photoelectron spectrum of pristine and LiP-0.1 NCM

Figure S4 Magnified FESEM images of (a,b) Pristine, (c,d) LiP-0.05, (e,f) LiP-0.1, (g,h) LiP-0.25 and (i,j) LiP-0.5

Figure S5 EDS mapping of LiP-0.1 showing elemental distribution of Ni, Co, Mn and P

Figure S1


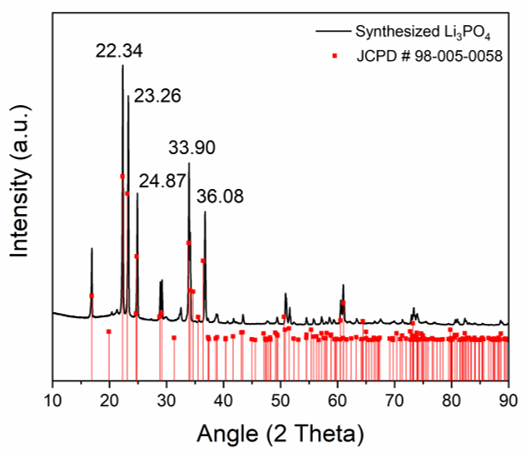


Figure S2


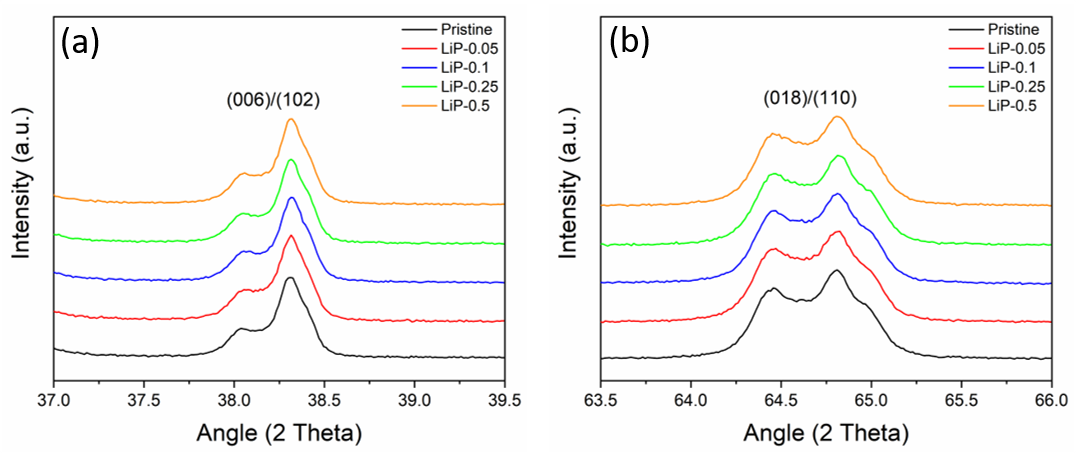


Figure S3


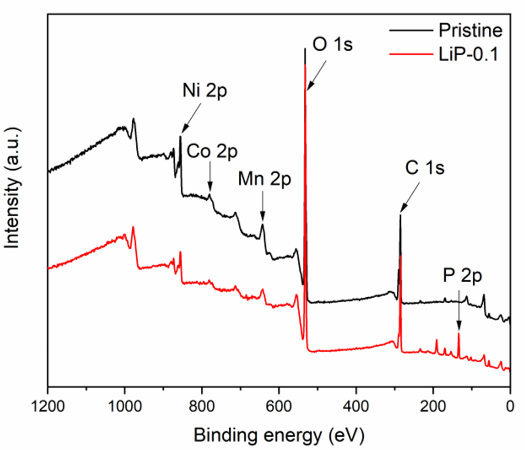


Figure S4


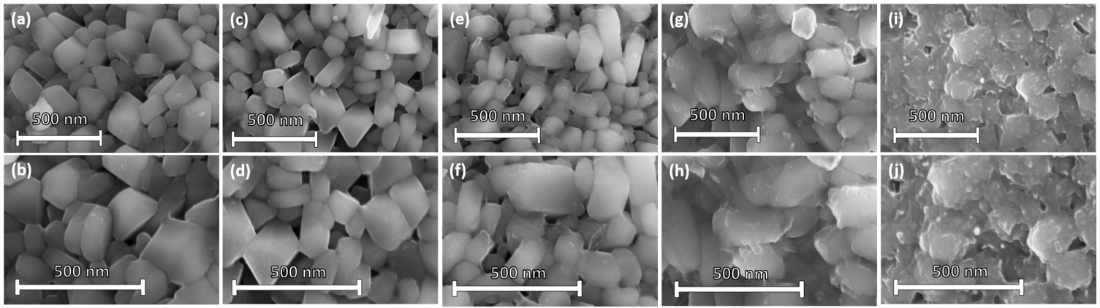


Figure S5


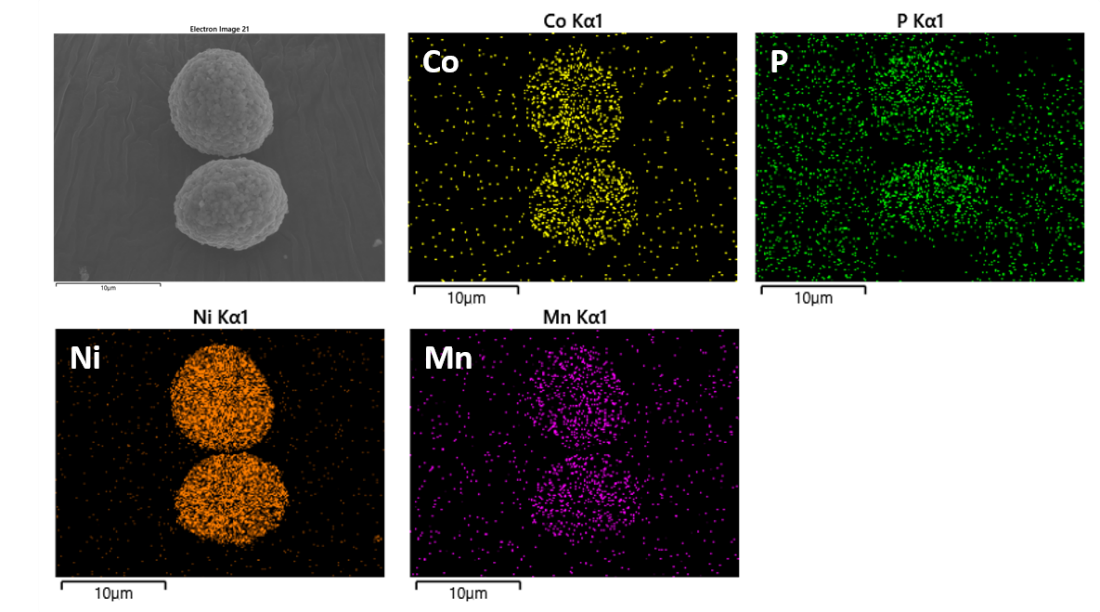

Supplement: Supplementary file 1 — Supplementary Information. [file 41598_2021_98123_MOESM1_ESM.docx]
